# Biomechanics and Inflammation in Atherosclerotic Plaque Erosion and Plaque Rupture: Implications for Cardiovascular Events in Women

**DOI:** 10.1371/journal.pone.0111785

**Published:** 2014-11-03

**Authors:** Ian C. Campbell, Jonathan D. Suever, Lucas H. Timmins, Alessandro Veneziani, Raymond P. Vito, Renu Virmani, John N. Oshinski, W. Robert Taylor

**Affiliations:** 1 Wallace H. Coulter Department of Biomedical Engineering, Georgia Institute of Technology/Emory University, Atlanta, Georgia, United States of America; 2 Cardiology Division, Department of Medicine, Emory University School of Medicine, Atlanta, Georgia, United States of America; 3 Department of Mathematics and Computer Science, Emory University, Atlanta, Georgia, United States of America; 4 George W. Woodruff Department of Mechanical Engineering, Georgia Institute of Technology, Atlanta, Georgia, United States of America; 5 CVPath Institute, Inc., Gaithersburg, Maryland, United States of America; 6 Department of Radiology and Imaging Science, Emory University, Atlanta, Georgia, United States of America; 7 Cardiology Division, Atlanta Veterans Affairs Medical Center, Decatur, Georgia, United States of America; Brigham and Women's Hospital, Harvard Medical School, United States of America

## Abstract

**Objective:**

Although plaque erosion causes approximately 40% of all coronary thrombi and disproportionally affects women more than men, its mechanism is not well understood. The role of tissue mechanics in plaque rupture and regulation of mechanosensitive inflammatory proteins is well established, but their role in plaque erosion is unknown. Given obvious differences in morphology between plaque erosion and rupture, we hypothesized that inflammation in general as well as the association between local mechanical strain and inflammation known to exist in plaque rupture may not occur in plaque erosion. Therefore, our objective was to determine if similar mechanisms underlie plaque rupture and plaque erosion.

**Methods and Results:**

We studied a total of 74 human coronary plaque specimens obtained at autopsy. Using lesion-specific computer modeling of solid mechanics, we calculated the stress and strain distribution for each plaque and determined if there were any relationships with markers of inflammation. Consistent with previous studies, inflammatory markers were positively associated with increasing strain in specimens with rupture and thin-cap fibroatheromas. Conversely, overall staining for inflammatory markers and apoptosis were significantly lower in erosion, and there was no relationship with mechanical strain. Samples with plaque erosion most closely resembled those with the stable phenotype of thick-cap fibroatheromas.

**Conclusions:**

In contrast to classic plaque rupture, plaque erosion was not associated with markers of inflammation and mechanical strain. These data suggest that plaque erosion is a distinct pathophysiological process with a different etiology and therefore raises the possibility that a different therapeutic approach may be required to prevent plaque erosion.

## Introduction

Thrombotic occlusion of the coronary arteries, a leading cause of morbidity and mortality worldwide, is caused by at least two distinct events. The most extensively studied phenomenon is plaque rupture, in which an advanced fibroatheroma develops through inflammation-mediated mechanisms and fissures, exposing the blood to the pro-thrombogenic necrotic core and yielding thrombus formation. More recently identified is a second major cause of sudden coronary death termed plaque erosion, where a thrombus forms that does not communicate with the necrotic core of an atherosclerotic plaque. Plaque erosion is a frequent event, occurring in up to 40% of fatal coronary thrombi [1], [2]. However, the demographics of individuals with the highest incidence of plaque erosion are noteworthy in that plaque erosion tends to occur in younger individuals, particularly women. It is estimated that plaque erosion may account for over 80% of coronary thrombi in women under age 50 [3]. These clinical differences raise the possibility of mechanistic differences between these etiologies of thrombotic occlusion of arteries.

Despite identification of plaque erosion as an important alternative mechanism for coronary thrombosis and a major cause of sudden death, its underlying pathophysiological mechanisms are not well understood. The leading mechanistic hypothesis for erosion (and its namesake) suggests that endothelial denudation of an atherosclerotic plaque exposes thrombogenic extracellular matrix to the blood. The cause of this focal denudation is not known, but possibilities include vasospasm and highly shearing flow [2] inducing endothelial apoptosis leading to denudation of the intimal surface of the vessel [4], [5]. Whereas plaque rupture is thought to be an inflammatory process, as cells like macrophages and lymphocytes that may induce apoptosis are prevalent in ruptured plaques, these same cells are not as widespread in eroded plaques [6]. Therefore, a better understanding of the factors leading to plaque erosion is needed.

Conversely, the mechanisms of plaque rupture have been extensively studied, and there is a well-accepted association between inflammation, plaque vulnerability, and biomechanics leading to atherosclerotic plaque instability and rupture. In addition to the biological mechanisms of inflammation and tissue remodeling, other factors affecting the stability of plaques include mechanical stress and strain. The association between biomechanics and plaque rupture is well established—a thin fibrous cap over a necrotic core usually has local maxima of stress on the cap or the plaque shoulders, sites where rupture is known to occur [7]. Additionally, mechanosensitive tissue markers related to inflammation and the development of atherosclerosis have been identified [8], [9]. Thus, mechanical forces in the vessel wall have been hypothesized to result in focal regions of inflammation and subsequent weakening of the fibrous cap. In addition, local inflammation may lead to strain heterogeneity, possibly resulting in plaque destabilization and rupture.

While it is well-established that inflammation and biomechanical forces within the arterial wall are essential components of plaque rupture, it's not clear if similar mechanisms are operative for plaque erosion. In this study, we investigated the roles of tissue biomechanics and inflammation in plaque disruption. We hypothesized that solid mechanical stresses and strains in the vessel wall are associated with inflammatory markers in plaque rupture but not in plaque erosion. These fundamental differences in biomechanics and subsequent inflammation could have broad implications not only for our greater understanding of the pathophysiology of atherosclerosis but perhaps, more importantly, for the selection of therapeutic strategies aimed at minimizing the clinical sequelae of atherosclerosis, particularly in younger women.

## Methods

### Overall Approach

Using the four standard plaque classifications: erosion, rupture, thin-cap fibroatheroma (TCFA, or “vulnerable” plaques), and thick-cap fibroatheroma, we quantified histological markers of atherosclerosis and inflammation that have known associations with plaque rupture. We used a histology-based, lesion-specific computational modeling technique to calculate the distribution of stresses and strains in the walls of atherosclerotic plaques and studied the spatial relationship between stress/strain and histologic markers.

### Human Tissue Histology

We obtained de-identified human coronary artery specimens from an existing tissue bank of hearts at CVPath Institute, Inc. (Gaithersburg, MD), obtained as a consultation service for the Maryland Office of the Chief Medical Examiner. Tissue harvest was described previously [1], [10]. Briefly, vessels were excised and perfusion-fixed with 10% neutral-buffered formalin at 100 mmHg for 15 minutes followed by dehydration and embedding in paraffin. We studied 74 specimens (33 erosion, 12 rupture, 15 TCFA, and 14 thick-cap fibroatheroma), and subject demographics are summarized in Table 1. Consecutive serial cross-sections for each subject were stained with Movat's pentachrome for lesion morphology and immunohistochemically with antibodies for CD68 for macrophages (Dako M0814), Factor VIII for endothelium (Strategic BioSolutions S40036NDI-D0), MMP1 for this collagenase (Enzo Life Sciences ADI-905-472), MMP9 for this elastase (Spring Bioscience E3660), a combination of CD31 and CD34 for vasa vasorum (Dako M0823 and Cell Sciences MON1164), smooth muscle actin for smooth muscle cells (Dako M0851), and TUNEL for apoptosis (Roche 12 156 792 910). Mosaic brightfield images of Movat's pentachrome and immunohistochemistry were acquired with a 4× objective and automatically stitched together using Microsoft Image Composite Editor (Microsoft Research, Redmond, WA). Confocal microscopy images of TUNEL staining were obtained with a 20× objective and automatically stitched together using Leica LAS AF (Leica Microsystems, Buffalo Grove, IL).

**Table 1:** Subject demographic information.

Case Type	n	Age (SD)	Gender	Race	Height (SD, in.)	Weight (SD, lbs.)	Hypertens. (%)	DM (%)	Smoker (%)
Erosion	33	43.5 (9.01)	25 M/8 F	22 W/11 B	68.5 (3.53)	196.3 (31.1)	6 (18.1%)	3 (9.09%)	4 (12.1%)
Thick-cap	14	51.2 (7.72)	10 M/2 F	9 W/3 B	70.6 (4.34)	224.8 (30.0)	1 (7.14%)	3 (21.4%)	3 (21.1%)
TCFA	15	58.5 (12.5)	11 M/4 F	10 W/4 B/1 Unknown	68.8 (4.19)	200.9 (53.2)	7 (46.7%)	2 (13.3%)	3 (20.0%)
Rupture	12	51.3 (12.0)	10 M/4 F	8 W/4 B/2 Other	68.9 (3.84)	194.4 (37.4)	8 (66.7%)	1 (8.33%	3 (25.0%)
Total	74	49.3 (12.2)	56 M/18 F	49 W/22 B/2 Other/1 Unknown	68.8 (3.67)	200.7 (40.6)	22 (29.7%)	9 (12.2%)	12 (16.2%)

For hypertension, diabetes mellitus, and smoker status, value is number of patients known to be a member of this cohort and may underestimate actual values. SD = Standard deviation, Hypertens. = hypertension, DM = diabetes mellitus, W = white, B = black.

### Ethics Statement

As this study was performed using cadaverous tissue, it does not constitute human subjects research. The Institutional Research Board at the Georgia Institute of Technology determined and documented that this research was classified as exempt from further IRB review.

### Image Segmentation

**Figure 1:**
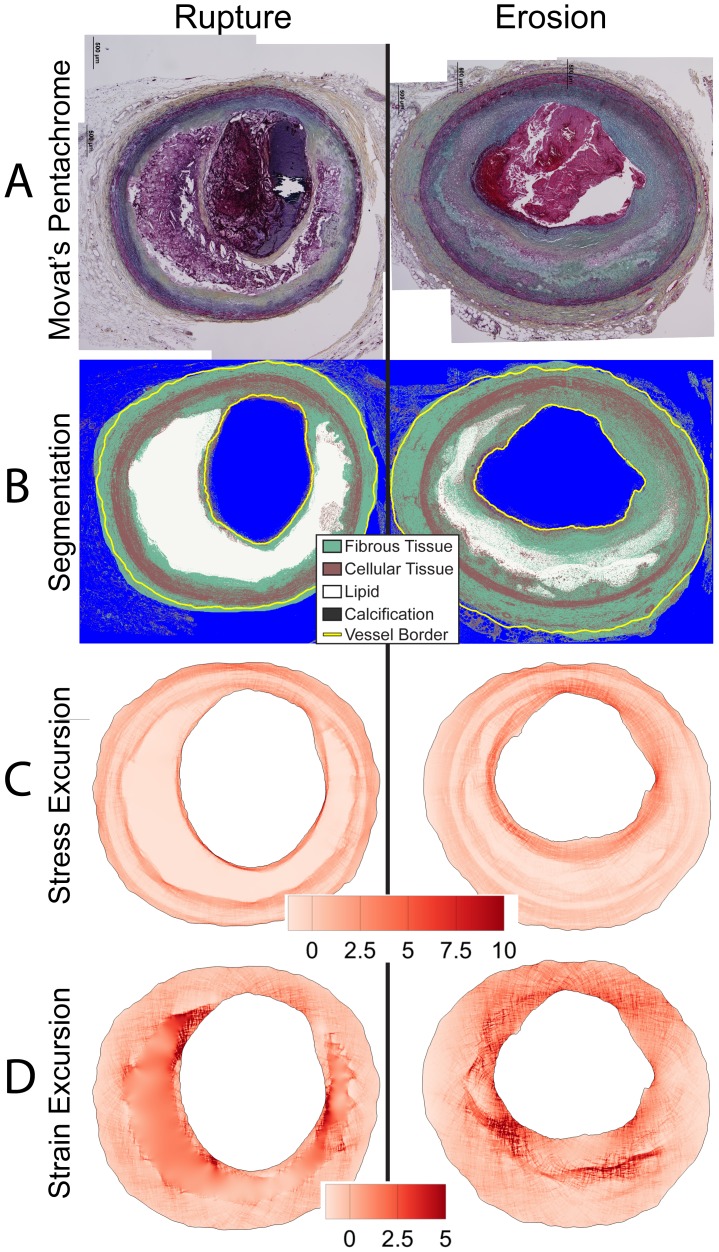
Histology segmentation and computational modeling. We segmented histologic cross-sections of human coronary arteries stained with Movat's pentachrome (A) in order to determine their composition (B) on a lesion-specific basis. Based on staining color, we identified whether tissues were fibrous, cellular, lipid/necrotic core, or calcified. We also traced the lumen and external border using an implementation of the Live Wire algorithm [12]. We computed and mapped the relative distribution of Von Mises stress and strain for each lesion. Shown here are stress (C) and strain (D) maps, displayed as excursion (normalized difference from the median).

**Figure 2:**
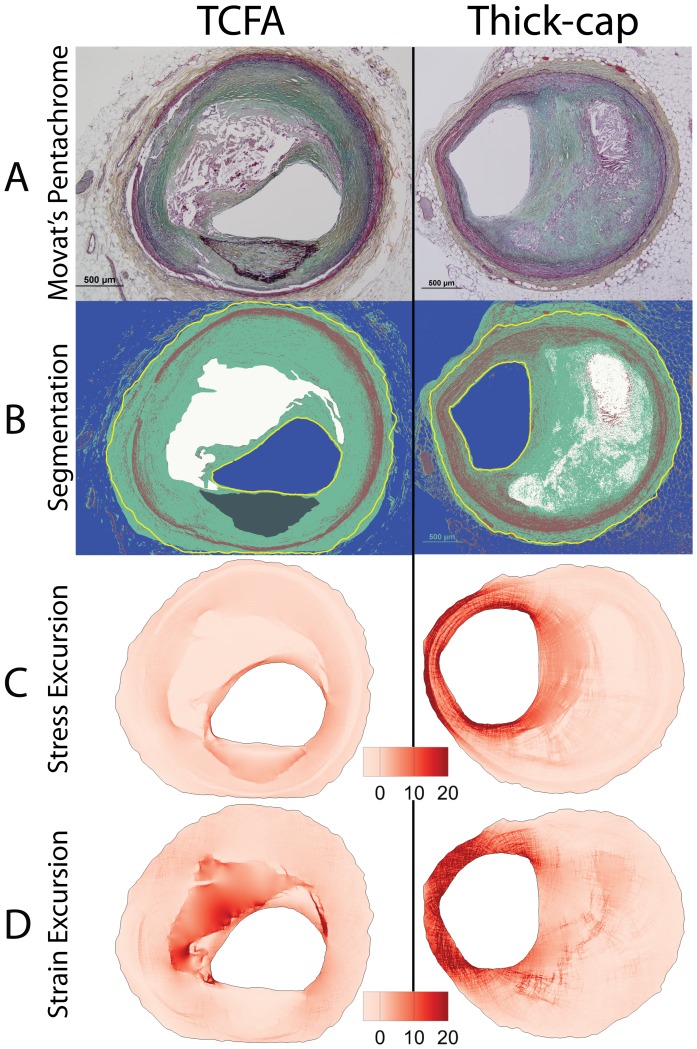
Representative images of thin-cap fibroatheroma (TCFA) and thick-cap fibroatheroma lesions. Movat's pentachrome images (A) were segmented (B), and maps of the relative distribution of stress excursion (C) and strain excursion (D) were created using identical techniques to those in Figure 1.

Movat's pentachrome images were automatically segmented to identify the composition and spatial distribution of tissue components using custom Matlab software (Figures 1–2 A, B). We used *k*-means clustering and Euclidean distance in LAB color space algorithms to identify the presence of fibrous, cellular, lipid/necrotic core, and calcified tissue based on staining color [11]. The *k*-means clustering approach automatically segmented each image based upon a statistical differentiation of pixel colors in RGB color space, given a desired number of color clusters to segment. When this approach provided a poor segmentation, the image was converted to LAB color space, an observer manually input a prototypical color for each stained tissue component, and the image was automatically segmented by computing the minimum distance between each pixel and the prototypical colors. An expert observer modified this segmentation as necessary around sectioning artifacts, mostly at the edges of calcium or necrotic core. Then, the lumen and periadventitial borders were traced using the Live Wire algorithm [12].

### Computational Modeling Approach and Assumptions

Based on this segmented map of tissue composition, we developed a lesion-specific computer model of the distribution of stresses in a 2-dimensional cross-section of each of the 74 vessels. We used an established finite element analysis modeling technique implemented in Ansys 14.0 software [8], [13] to calculate the relative distribution of Von Mises stress and strain in each vessel (Figures 1–2 C, D). We examined mechanical parameters on a relative rather than absolute scale due to absence of residual stress data in pressure-fixed tissue. Therefore, to compare values between lesions, we computed the stress and strain excursion, defined as the spatially-resolved percent difference of stress or strain from the lesion's median value.

Linear, elastic, isotropic material properties were used for all tissue components [14]. Lipid/necrotic core elastic modulus was 3.875×10^4^ Pa, cellular tissue was 2.449×10^5^ Pa, fibrous tissue was 1.821×10^6^ Pa, and calcified tissue elastic modulus was 1.066×10^7^ Pa. Although biological tissues are known to be nonlinear, viscoelastic, and anisotropic [15]–[20], assignment of such material properties to pressure-fixed tissue is mathematically complex and requires a battery of simplifying assumptions. For example, properly applying anisotropic material properties in a subject-specific model of a complex lesion requires knowledge of fiber directions that we do not have, and therefore we assume isotropic material properties.

Material properties of vascular tissues can be approximated with a bilinear stress-strain curve, with a break point between the two realms occurring slightly below our fixation pressure. Below this threshold, very little stress is generated as the vessel inflates. Therefore, although we are unable to calculate the stresses in the wall from blood pressures below diastole, we assume these stresses are relatively low. The relative contribution from stresses above diastolic pressure is much greater (and is the basis for our model), which is why we can use our technique to determine the relative distribution of high and low stress and strain but not the exact magnitude.

To model each vessel, we generated a fine-resolution finite element mesh of several hundred thousand elements between the lumen and periadventital boundary. For each element, we used a rule-of-mixtures approach [14], [20] to estimate the elastic modulus for that element, based on our segmented pentachrome image. We then simulated an incremental pressure of +40 mm Hg (approximately equal to the difference between diastole, the pressure at which the vessel was fixed, and systole) normal to the lumen. The resulting Von Mises stress and strain distributions for each vessel were generated, representing the distribution of stresses in the lesion at peak systole.

### Artifact Removal

**Figure 3:**
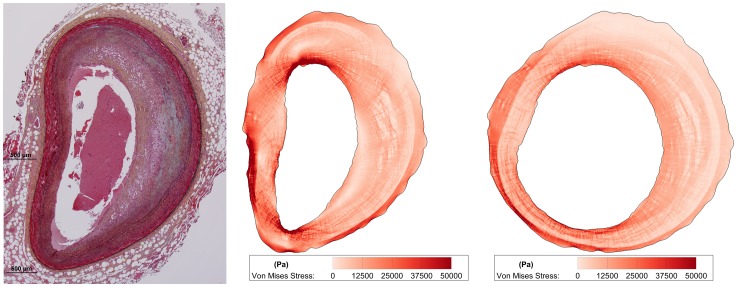
Preinflation technique for mechanical modeling. Many histology specimens suffered from fixation and embedding artifacts, such as the vessel at left, which is horizontally compressed relative to its *in vivo* conformation where the lumen would be mostly circular. Consequently, the computed stress and strain distributions were skewed in regions of high deformation during computational analysis (middle). Therefore, we “pre-inflated” vessels by simulating pressure normal to the lumen, allowing the vessel to deform, and re-meshing the vessel for 10 iterations. This extra processing step minimized the effect of tissue artifacts and presented us with more physiologically-relevant anatomy for our analysis (right).

Some vessels suffered from histology artifacts such as compression along one axis. These artifacts were apparent because the lumen was not approximately circular (Figure 3, left), as would be the case *in vivo* in a pressurized vessel. The consequence of this artifact in our mechanical model was an artificially-high region of both stress and strain at sites of highest deformation (Figure 3, middle). These obscured the physiological stress and strain distribution, and therefore, we digitally “pre-inflated” each vessel to allow it to distend into a more natural starting conformation. For 10 computer modeling iterations, we simulated a +40 mm Hg incremental pressure normal to the lumen, computed the new conformation of the vessel, remeshed the vessel to overcome highly-skewed elements, and interpolated new material properties for each element based upon the previous iteration's deformed spatial map of material properties. The final iteration of this stepped process was used for our analysis (Figure 3, right) and did not exhibit local stress concentrations at sites of histology artifacts. Sensitivity analysis on the number of iterations revealed that after 10 steps, results were not significantly affected (percent change in stress and strain excursion was typically <1% between iteration 10 and subsequent iterations). We examined the stress and strain excursion distributions (similar to Figure 1C–D) and their association with positive histological staining for 3 randomly-selected sections (including those both with and without artifacts) from each of the four plaque phenotypes and observed that results changed only trivially between 10, 15, and 20 iterations.

### Morphology and Composition

We calculated the relative composition of each lesion phenotype from image segmentation. Several geometric parameters were evaluated to determine if the shape of lesions differed by phenotype. We computed plaque burden (percent of area inside internal elastic lamina [IEL] containing lesion), lesion cross-sectional area (area of tissue inside the IEL), vessel external radius (radius of the smallest possible circle which contained each vessel), vessel eccentricity (distance between lumen centroid and IEL centroid, normalized to vessel radius), and the average thickness of the media (average distance between the inner and outer boundaries of the media, where present) [21]. Data are presented in Table S1.

### Mechanics and Tissue Marker Analysis

To evaluate if mechanical stress and strain was associated with positive staining for each of the 7 tissue markers, we registered each stained image against the segmented pentachrome image that was the basis for mechanical modeling. The lumen in each image was traced, and rigid registration was performed using cross-correlation of the distance from the lumen border to its centroid to determine the necessary degree of rotation [21], [22]. Regions exhibiting histology artifacts were locally excluded from analysis. Images were thresholded to identify positive staining using empirically-determined color levels. For each element of the fine-resolution mesh used for computational mechanics, we determined the stress and strain excursion and, as a binary metric, whether positive staining was present.

**Figure 4:**
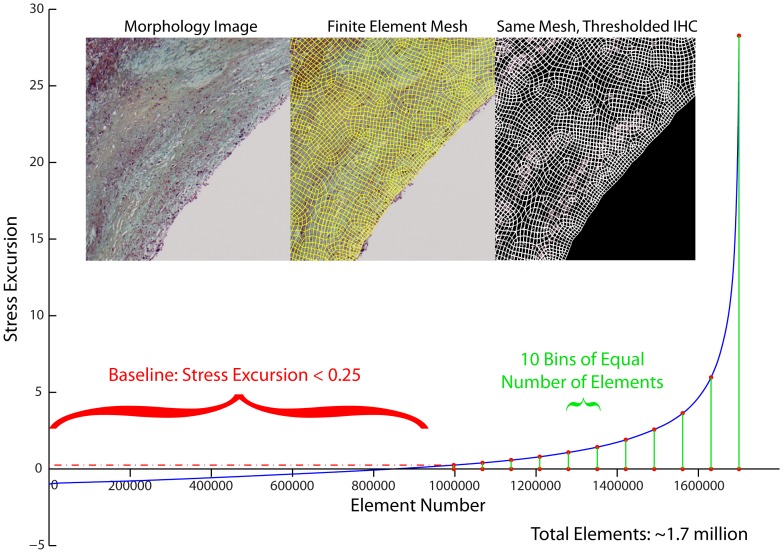
Stress and strain excursion association with positive staining. Von Mises stress and strain excursions were computed for each lesion by breaking down each vessel into a fine-resolution mesh. After sorting the elements in order of increasing stress (or strain) excursion, we pooled results into a baseline group (excursion <0.25) and tertiles or deciles containing an equal number of elements. Thus, higher-numbered groups (Figures 5–8) represent regions of higher stress or strain excursion. Negative stress excursion represents stress below the median value, not a negative magnitude of stress.

To determine if there was an association between stress/strain excursion and each stain, we pooled results within each of the four lesion phenotypes and sorted them in order of ascending stress or strain excursion (Figure 4). We then divided each phenotype into a baseline group with excursion <0.25, and then tertiles and deciles of equal-number elements [8]. Both tertiles and deciles were considered to ensure that any associations detected were not dependent on group size.

### Staining

To evaluate the total expression of each marker per vessel, we thresholded each stained image using the same empirically-determined color levels as in the mechanical analysis and calculated the percent of each vessel with positive staining. Results were tallied for each phenotype (Table S2).

### Endothelial Denudation and Apoptosis

To determine the extent of endothelial apoptosis and denudation, we quantified the amount of positive staining for Factor VIII and TUNEL within 10 µm of the lumen border. We manually traced the lumen of each image then dilated the contour 10 µm using a signed distance function. We tallied the percent of positive pixels within this region for each phenotype. Local regions of sections with significant thrombus adherent to the wall were excluded because we could not differentiate between positive staining from endothelium vs. thrombus. Factor VIII was selected as our endothelial marker because its abundance in these cells ensures we would not underestimate the amount of endothelium present.

### Box Plots

Summary data of positive staining, morphology, and vessel composition were summarized in box plots using the definition of Tukey. The bold center line represents the median value, and the horizontal box limits represent the 25^th^ and 75^th^ percentiles of the data. Whiskers extend to 1.5 times the interquartile range, and outlier points are represented by dots. Notches represent the 95% confidence interval for each median.

### Statistical Analysis

To evaluate differences between tertiles and deciles in our mechanics and histology analysis, we performed a chi-squared analysis to determine if an association existed. If so, we subsequently performed a two-proportion *post hoc* test to compare each tertile or decile to its baseline with Bonferroni correction for 11 comparisons between deciles with α = 0.05.

For total staining, endothelial denudation and apoptosis, and morphology and composition analyses above, statistical differences between phenotypes were assessed with one-way ANOVA followed by Tukey's honestly significant difference *post hoc* test with Bonferroni correction for 6 permutations of comparisons between phenotypes with α = 0.05.

## Results

### Mechanics

We successfully segmented 33 eroded plaques, 12 ruptured plaques, 15 TCFA, and 14 thick-cap fibroatheromas (Figures 1–2) and calculated lesion-specific distributions of Von Mises stress and strain for each. Local maxima of stress excursion tended to be concentrated on fibrous caps over necrotic cores. In ruptured and TCFA plaques (both phenotypes with thin fibrous caps), these local maxima were highly focal, whereas in eroded and thick-cap fibroatheroma specimens with relatively thicker fibrous caps, the local maxima of stress excursion were more diffuse across larger areas. We identified no qualitative difference in distribution of strain excursion between the four phenotypes. Additionally, there was no significant difference in computed median stress or strain between any of the groups, as assessed by Tukey's *post-hoc* test (p>0.58 for all groups).

### Mechanics and Markers of Inflammatory Pathology

**Figure 5:**
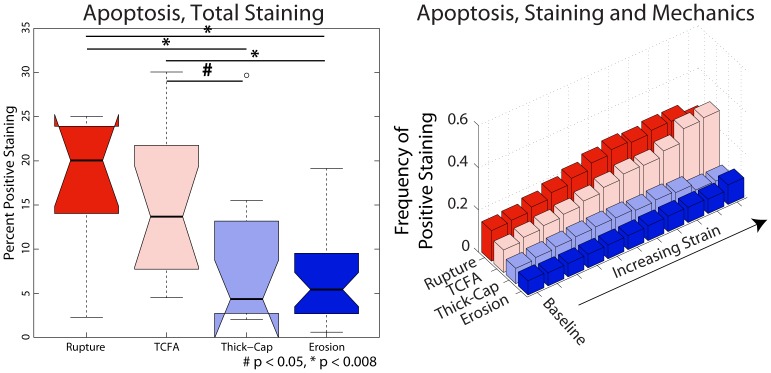
Apoptosis expression depends on both lesion phenotype and mechanical strain. TUNEL staining for apoptosis (Figure 9) expression across the entire vessel cross-section is significantly lower in plaque erosions and thick-cap fibroatheromas (stable plaques) than in ruptured and TCFA plaques (left). Whereas sites with elevated mechanical strain are positively associated with the presence of apoptosis in ruptured and TCFA (vulnerable) plaques, there is no positive association between strain and apoptosis in plaque erosions and thick-cap fibroatheromas (right).

**Figure 6:**
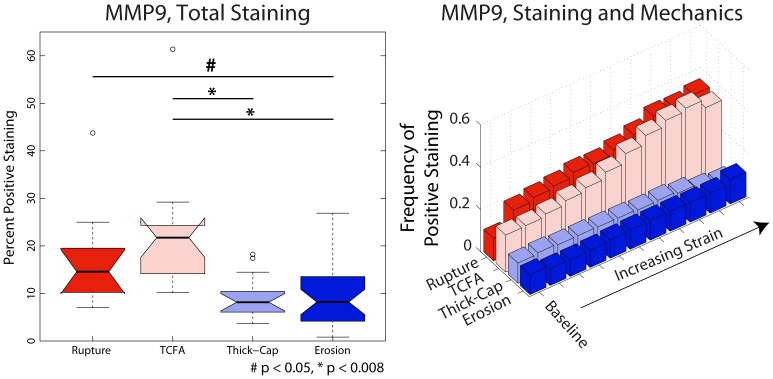
MMP9 expression depends on both lesion phenotype and mechanical strain. MMP9, a gelatinase, expression across the entire vessel cross-section (Figure 10) is significantly lower in plaque erosions and thick-cap fibroatheromas (stable plaques) than in TCFA plaques vulnerable to rupture (left). Whereas sites with elevated mechanical strain are positively associated with the presence of MMP9 in ruptured and TCFA (vulnerable) plaques, there is no positive association between strain and MMP9 in plaque erosions and thick-cap fibroatheromas (right).

**Figure 7:**
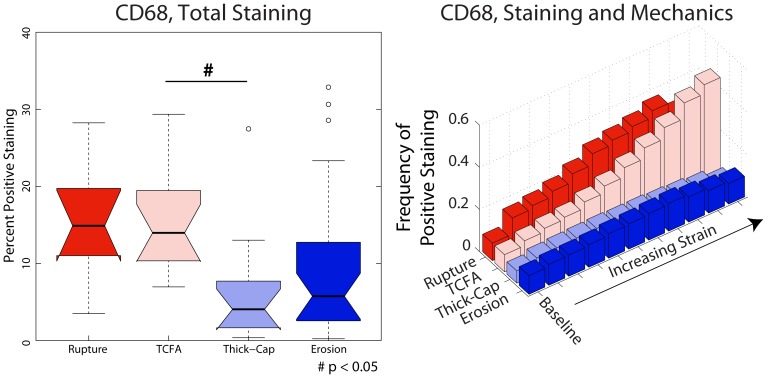
CD68 expression depends on both lesion phenotype and mechanical strain. CD68, a marker of the macrophages that play a major role in lesion inflammation (Figure 11), median expression across the entire vessel cross-section is lower in plaque erosions and thick-cap fibroatheromas (stable plaques) than in plaques vulnerable to rupture (left). Whereas sites with elevated mechanical strain are positively associated with the presence of macrophages in ruptured and TCFA (vulnerable) plaques, there is no positive association between strain and macrophages in plaque erosions and thick-cap fibroatheromas (right).

**Figure 8:**
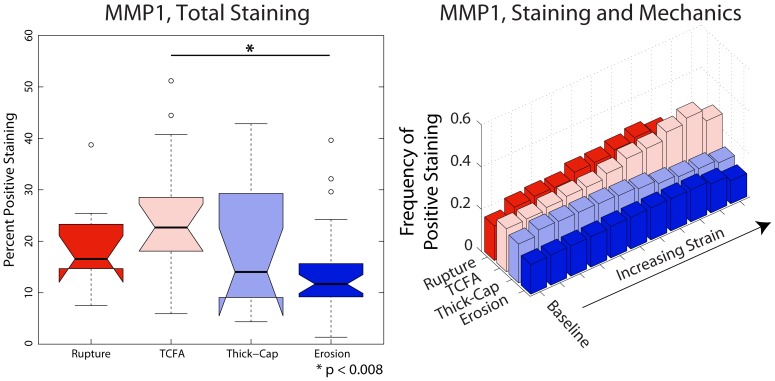
MMP1 expression depends on both lesion phenotype and mechanical strain. Expression of MMP1, a collagenase, across the entire vessel cross-section (Figure 12) is significantlylower in plaque erosions than in plaques vulnerable to rupture (left). Whereas sites with elevated mechanical strain are positively associated with the presence of macrophages in ruptured and TCFA (vulnerable) plaques, there is no positive association between strain and macrophages in plaque erosions and thick-cap fibroatheromas (right).

We identified phenotype-dependent differences in expression of inflammatory markers and their association with mechanical strain in the different plaque types (Figures 5–8). First, we quantified the percentage of each lesion exhibiting positive staining for immunohistochemical markers (left panels, Figures 5–8) and found that, in general, ruptured and TCFA plaques had higher mean and median values of percent staining for inflammatory markers than eroded plaques and thick-cap fibroatheromas. Specifically, eroded plaques exhibited lower mean staining for TUNEL (Figure 5) and MMP9 (p<0.008; Figure 6) across the entire section than ruptured and TCFA plaques, and erosions had significantly lower mean staining for MMP1 than TCFA plaques (Figure 8). Plaque erosions were not significantly different from thick-cap fibroatheroma (stable) plaques for any immunohistochemical marker, and plaque ruptures were not significantly different from TCFAs for any marker.

**Figure 9:**
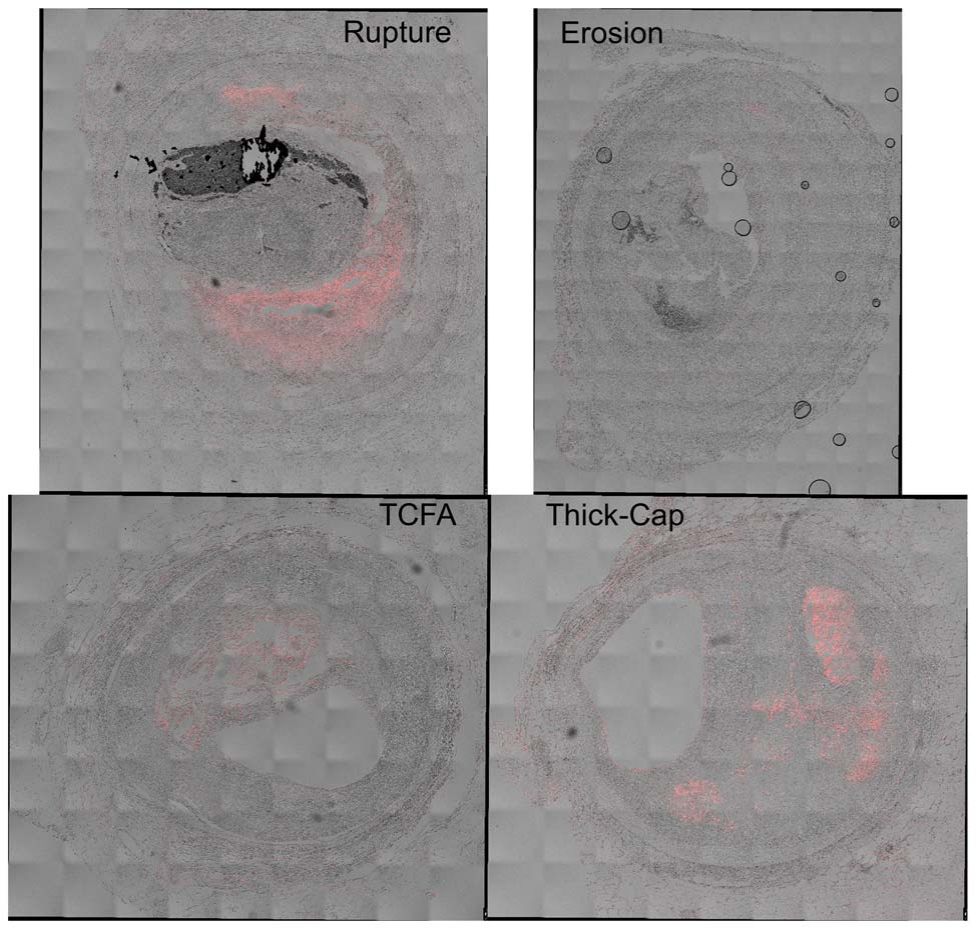
Representative histology images of TUNEL staining. Serial sections of the lesions shown in Figures 1 and 2 were stained for apoptosis using the TUNEL approach (red staining), and differential interference contrast (DIC) is shown for reference. Regions containing coverslip bubbles such as that seen in the erosion specimen were manually masked and excluded from final analysis.

**Figure 10:**
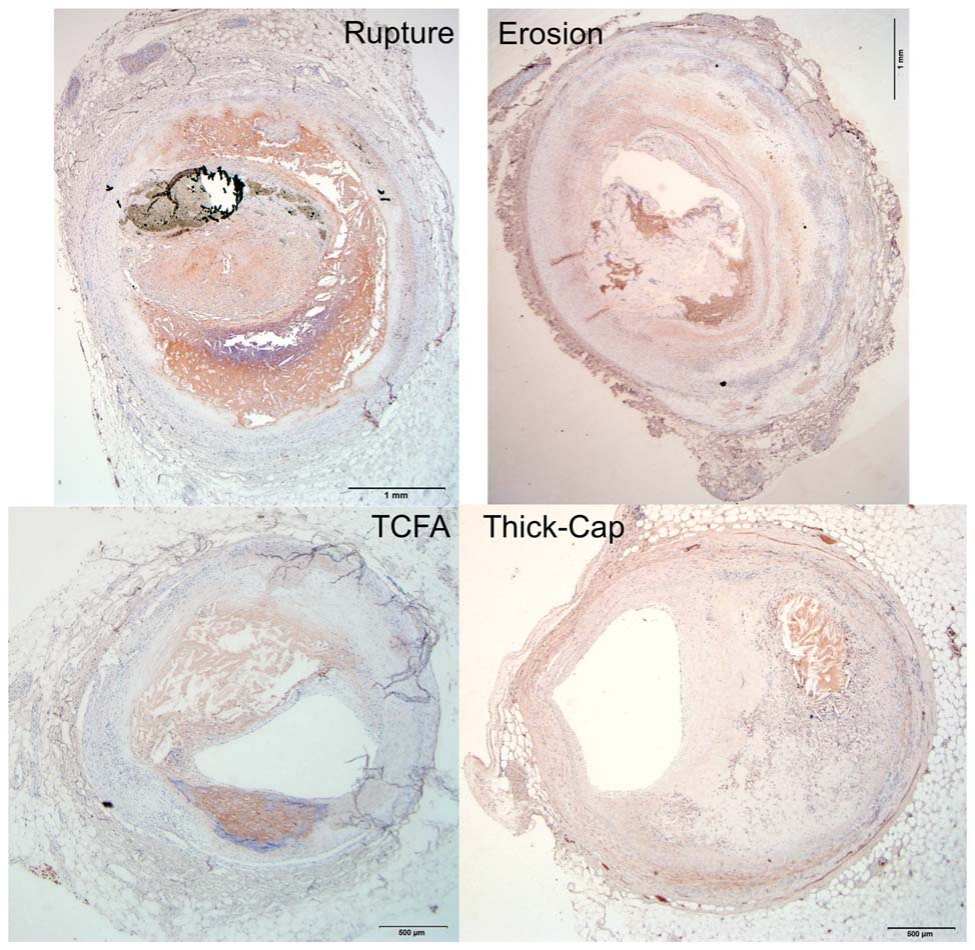
Representative histology images of MMP9 staining. Serial sections of the lesions shown in Figures 1 and 2 were stained for MMP9 (Nova Red chromagen) and counterstained with hematoxylin (blue) for nuclei. Artifacts such as the tissue detachment seen in the TCFA specimen were manually masked to exclude the regions from the final analysis.

**Figure 11:**
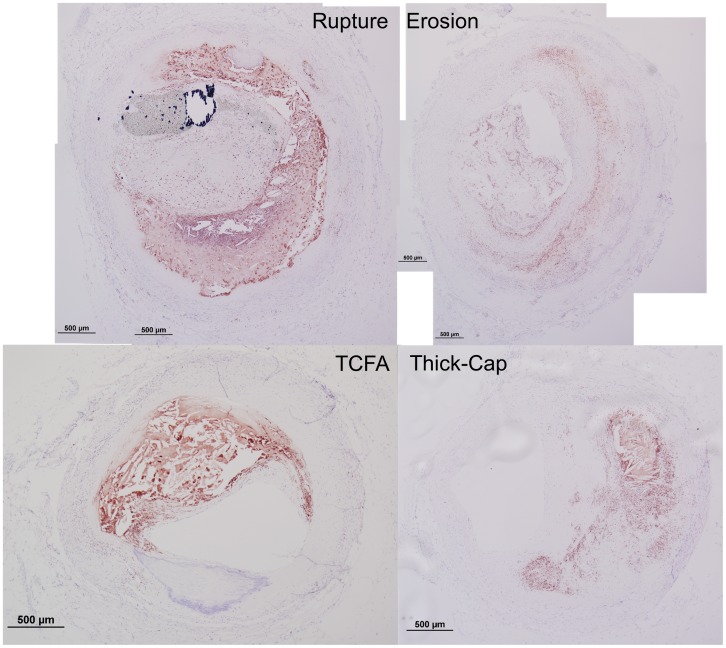
Representative histology images of CD68 staining. Serial sections of the lesions shown in Figures 1 and 2 were stained for CD68 (Nova Red chromagen) and counterstained with hematoxylin (blue) for nuclei.

**Figure 12:**
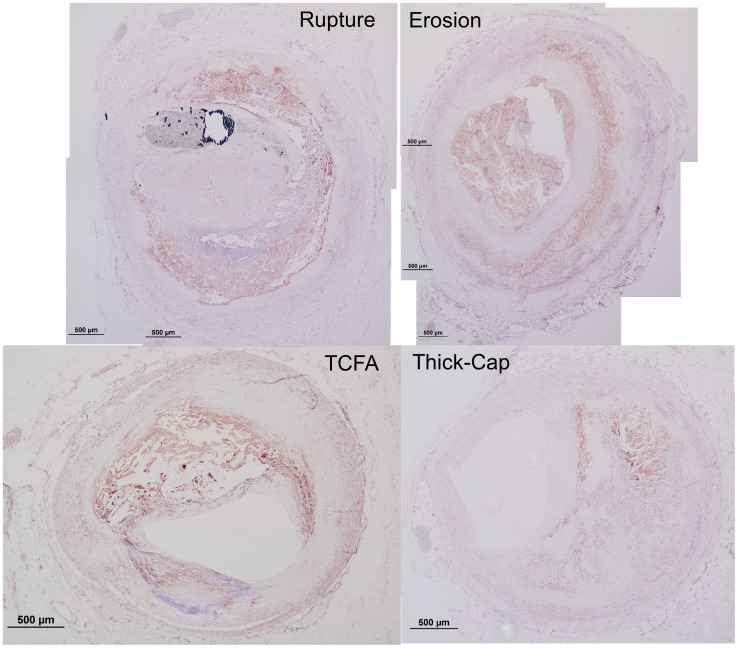
Representative histology images of MMP1 staining. Serial sections of the lesions shown in Figures 1 and 2 were stained for MMP1 (Nova Red chromagen) and counterstained with hematoxylin (blue) for nuclei.

Next, we examined how the spatial locations of positive staining for immunohistochemical markers of inflammation (representative histological samples, Figures 9–12) were associated with the distribution of tissue mechanical stress and strain. This analysis was motivated by the concept that tissue mechanics can regulate biochemical function through the process of mechanotransduction and the hypothesis that the role of mechanics may vary in different plaque phenotypes. The complex heterogeneous composition of atherosclerotic lesions leads magnitudes of mechanical stress and strain that vary spatially across the lesion cross-section. By registering consecutive immunohistochemical serial sections of lesions to the computed distribution of stress and strain, we can analyze associations between inflammation and relative magnitude of mechanical parameters using histogram analysis, as summarized in Figure 4.

**Figure 13:**
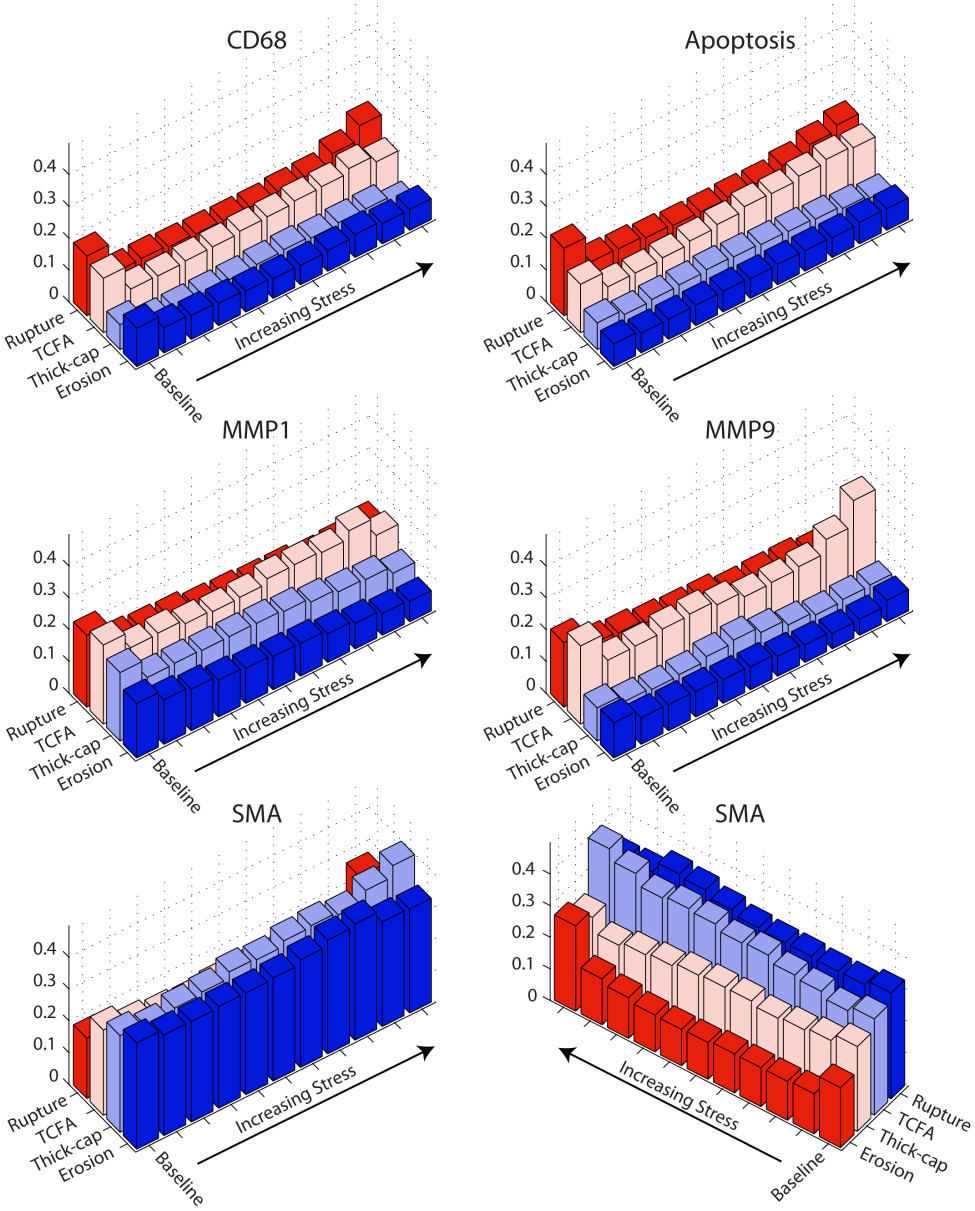
Positive staining and mechanical stress. We examined positive staining for inflammatory markers in images registered against Von Mises stress maps (Figure 1C). We divided stress excursion into a baseline group (excursion <0.25) and deciles of equal number of elements (Figure 4) then examined the frequency of positive staining associated with each level of mechanical strain.

When tissue mechanics were taken into account, markers of inflammation and apoptosis (CD68, MMP1, MMP9, and TUNEL) were approximately uniformly distributed among all levels of strain in samples identified as erosion and thick-cap fibroatheroma, (right panels, Figures 5–8). All bins in the histogram corresponding to these plaque phenotypes are nearly the same height regardless of strain. In contrast, markers of inflammation and apoptosis were positively associated with greater magnitudes of mechanical strain in ruptured and TCFA plaques. Histogram bin heights increased with mechanical strain for these plaque phenotypes. The characteristics of plaques with erosion more closely resembled thick-cap fibroatheromas, the more stable phenotype. Mechanical stress, as opposed to strain, was not associated with these markers for any phenotype (Figure 13).

### Morphology and Composition

**Figure 14:**
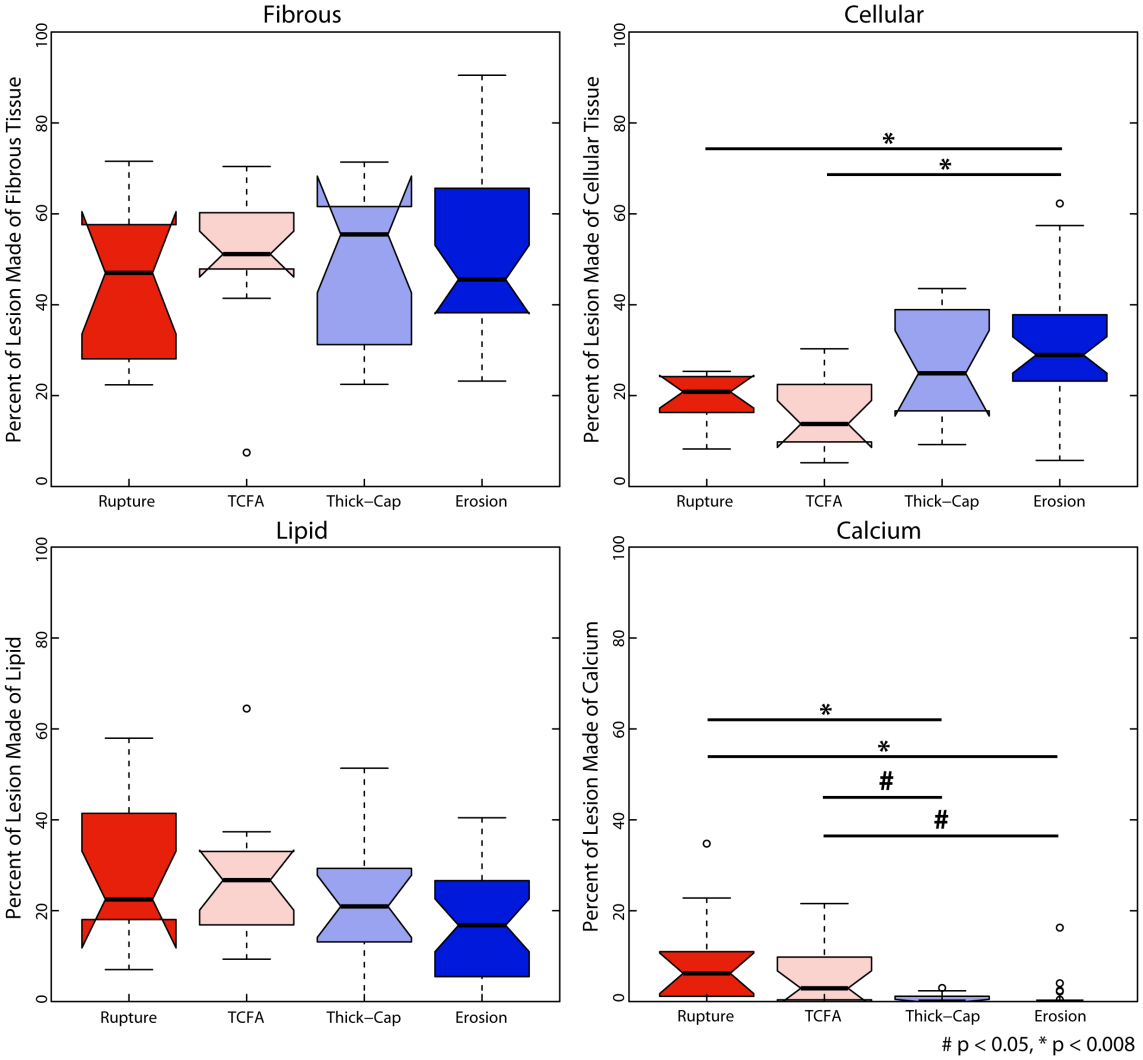
Relative composition. Based on image segmentation (Figure 1), we calculated the relative composition of lesions for each of the four phenotypes. Plaque erosion specimens were significantly more cellular and less calcified than ruptured plaques.

**Figure 15:**
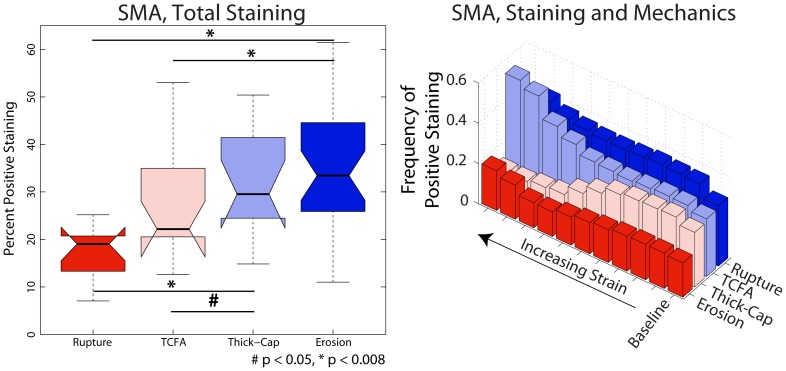
SMA expression depends on both lesion phenotype and mechanical strain. SMA exhibited a trend opposite inflammatory markers: it varied positively with increasing strain excursion for eroded and thick-cap fibroatheroma plaques (Figure 4), negatively for TCFA plaques, and had no dominant association for ruptured plaques. Across the entire vessels (Figure 16), plaque erosions had significantly more smooth muscle than did ruptured plaques (p<0.008). Representative images are shown in Figure 12.

**Figure 16:**
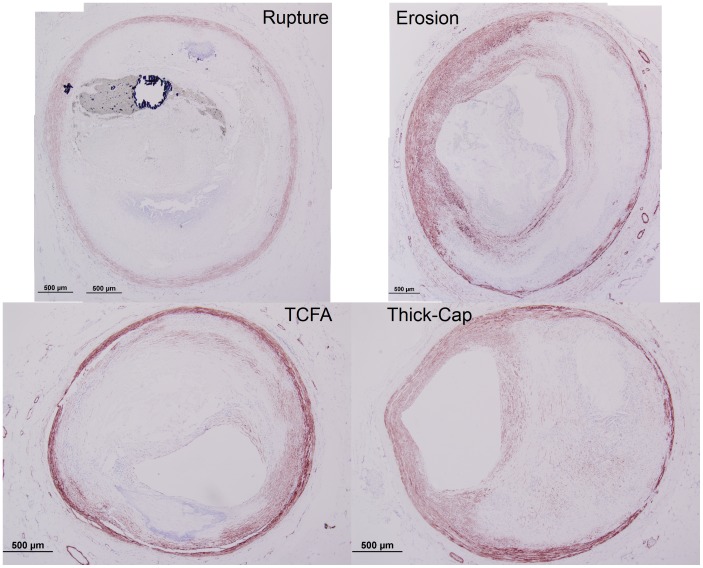
Representative histology images of smooth-muscle actin staining. Serial sections of the lesions shown in Figures 1 and 2 were stained for smooth muscle actin (Nova Red chromagen) and counterstained with hematoxylin (blue) for nuclei.

**Figure 17:**
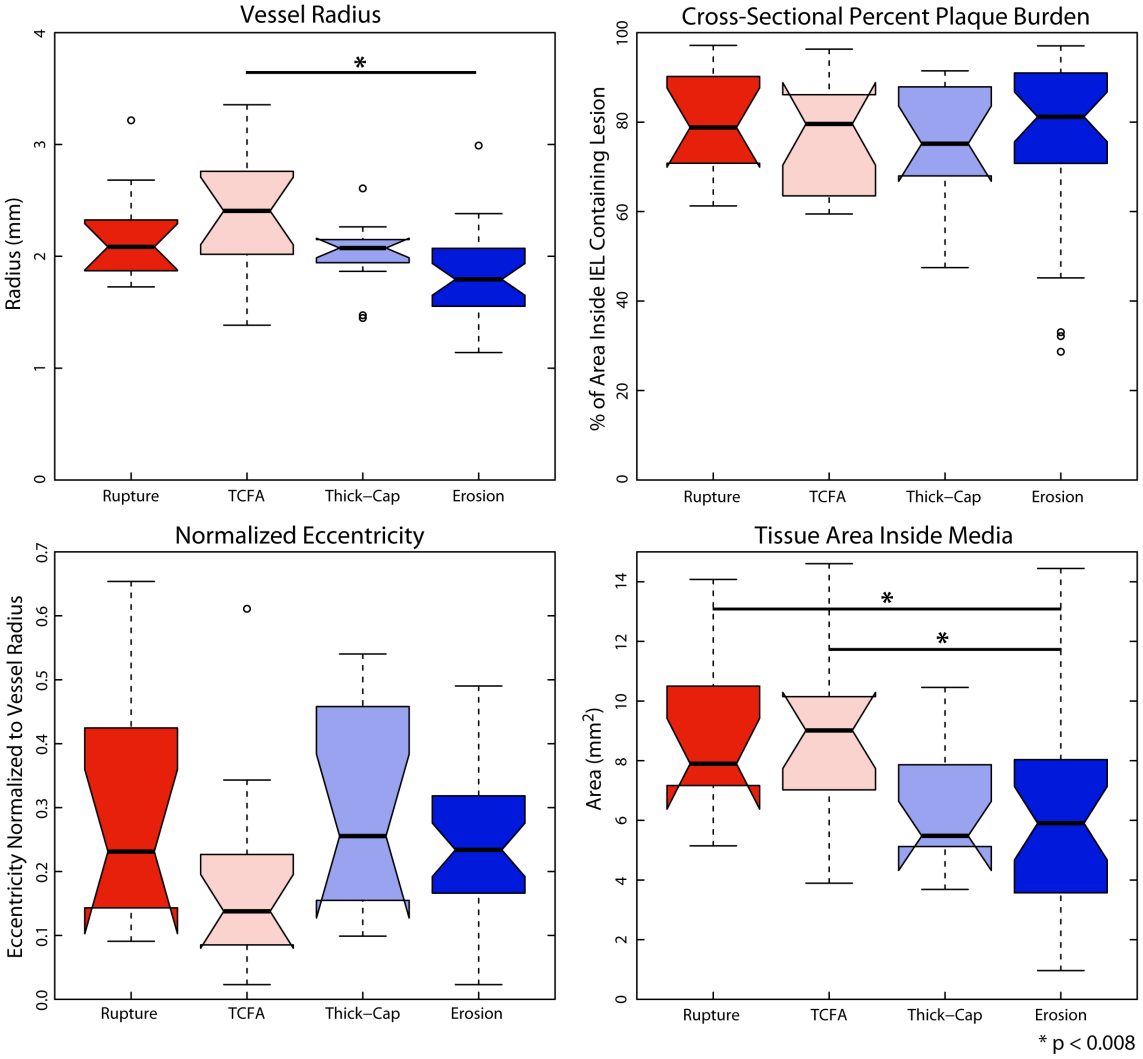
Morphological metrics. We calculated vessel radius (by circumscribing a circle around each vessel, upper left), the plaque burden (the percent of the area inside the IEL containing lesion, upper right), the eccentricity (distance between lumen centroid and IEL centroid, normalized to vessel radius, lower left) and area containing tissue inside the IEL (lower right). Differences between phenotypes were not significant, except for radius between erosion and TCFA and that erosions had significantly smaller cross-sectional area than ruptured plaques and TCFAs.

Plaque erosions contained significantly more cellular tissue than ruptured or TCFA plaques but were not different from thick-cap fibroatheroma plaques (Figure 14), and an identical trend was observed for positive staining for SMA (p<0.008, Figures 15–16). Erosions and thick-cap fibroatheromas also had significantly less calcium than ruptured plaques (p<0.008) and had less calcium than TCFAs (p<0.05). Erosions had significantly smaller external radii than TCFA plaques (p<0.008) and had significantly smaller cross-sectional area (p<0.008) than ruptured and TCFA plaques (Figure 17). Despite differences in cross-sectional areas of plaques between phenotypes, there was no difference in the relative proportion of lesion composed of fibrous tissue or lipid/necrotic core between any of the phenotypes. There was no difference in cross-sectional percent plaque burden or eccentricity between lesions, but the cross-sectional area of eroded plaques was less than ruptured or TCFA plaques (p<0.05).

### Endothelium

**Figure 18:**
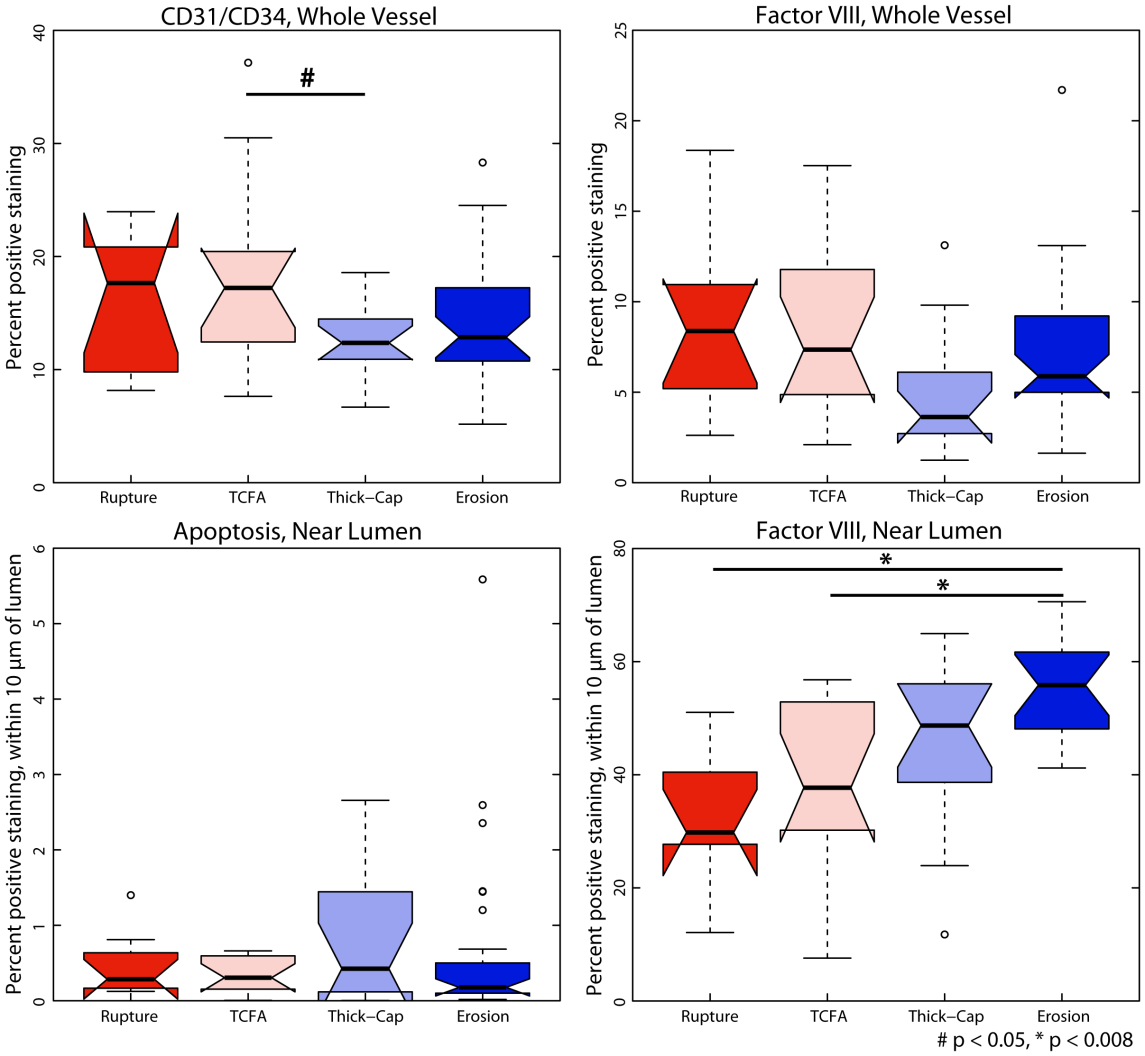
Apoptosis and Factor VIII for whole vessel and near lumen. Plaque erosion has been hypothesized to result from endothelial denudation, possibly resulting from apoptosis. When examining the whole vessel, eroded plaques are significantly less apoptotic (Figure 5) than ruptured plaques or TCFAs but no significant difference in total Factor VIII and CD31/CD34. When isolating the endothelium (by examining a region within 10 µm of the lumen) eroded plaques did not have a significantly greater number of apoptotic cells and had significantly more Factor VIII positive cells than ruptured plaques or TCFAs.

**Figure 19:**
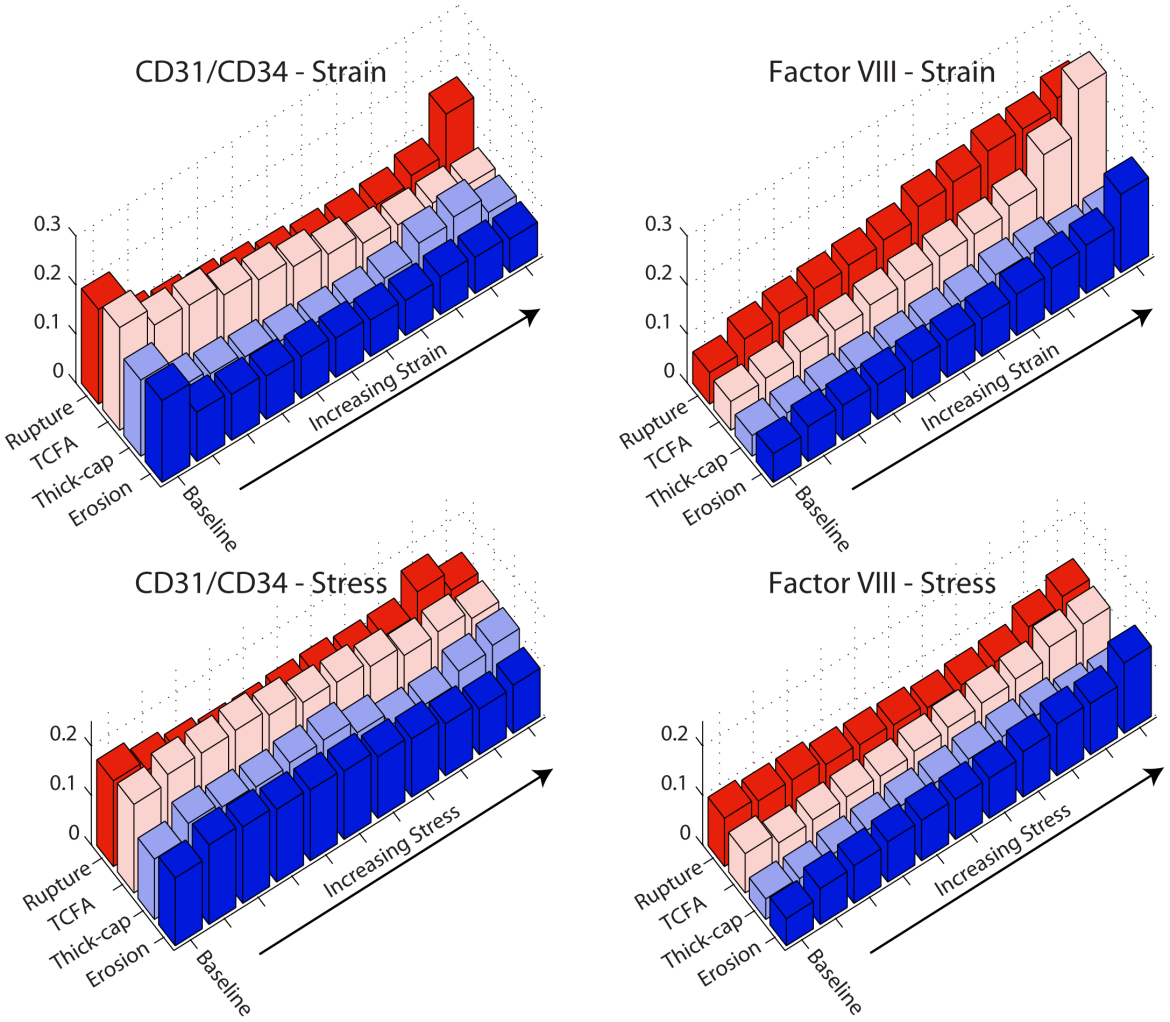
Positive staining for endothelial markers and mechanical stress and strain. We examined positive staining for endothelial markers in images registered against Von Mises stress and strain maps (Figure 1C–D). We divided stress excursion into a baseline group (excursion <0.25) and deciles of equal number of elements (see Figure 4) then examined the frequency of positive staining associated with each level of mechanical strain.

**Figure 20:**
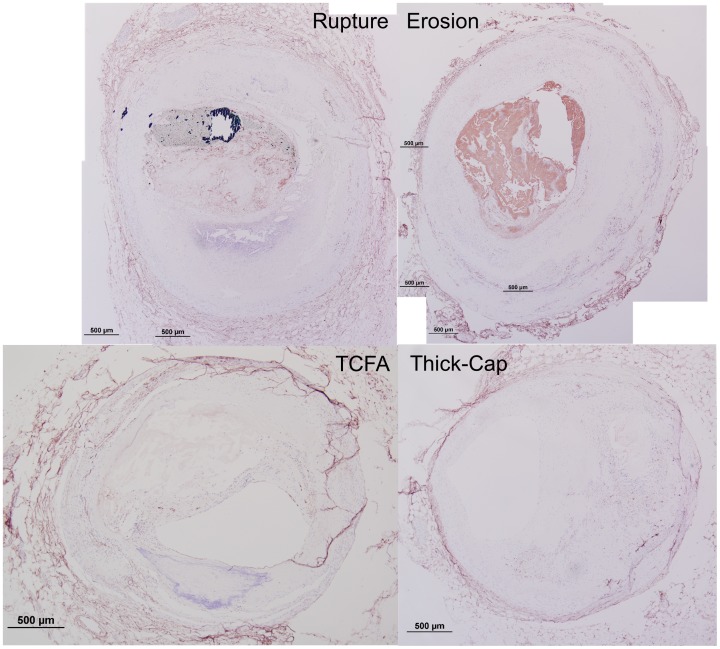
Representative histology images of CD31/CD34 staining. Serial sections of the lesions shown in Figures 1 and 2 were stained for a combination of CD31 and CD34 (Nova Red chromagen) and counterstained with hematoxylin (blue) for nuclei. Artifacts such as the adventitial detachment in the TCFA specimen were manually masked out and excluded from the final analysis.

**Figure 21:**
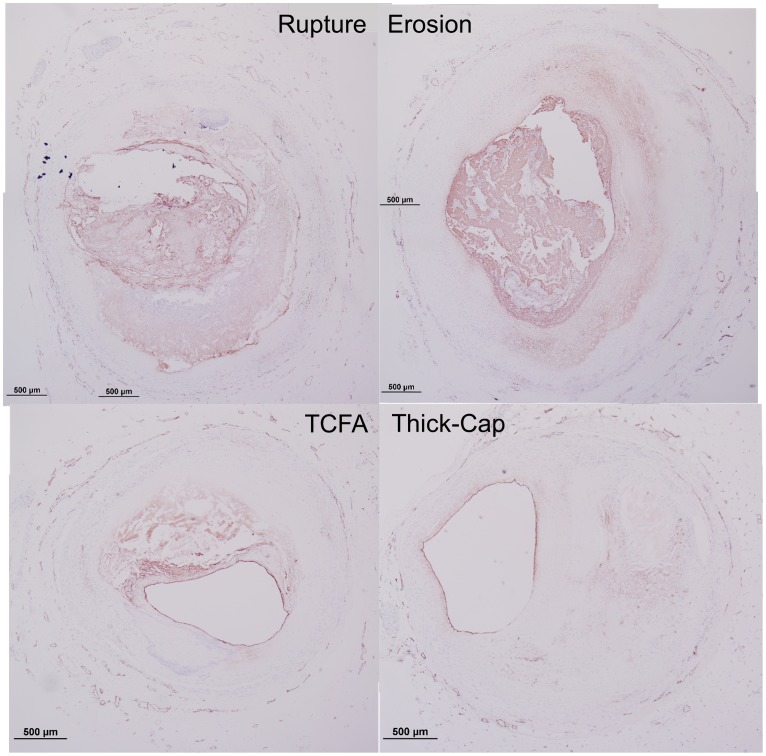
Representative histology images of Factor VIII staining. Serial sections of the lesions shown in Figures 1 and 2 were stained for Factor VIII (Nova Red chromagen) and counterstained with hematoxylin (blue) for nuclei.

Given that previous studies have suggested a role for endothelial denudation and apoptosis in plaque erosion, we quantified apoptosis with TUNEL staining. There was no significant difference in endothelial apoptosis between the four phenotypes of plaques. Somewhat surprisingly, there were significantly more (p<0.008) Factor VIII-positive cells in plaque erosion than in ruptured or TCFA plaques (Figure 18). Eroded plaques were not significantly different from thick-cap fibroatheroma plaques. Across the entire vessel, there was no outstanding association for either of the endothelial markers CD31/CD34 or Factor VIII with stress/strain for any phenotypes (Figure 19). Representative histology specimens are shown in Figures 20–21.

## Discussion

In this study, we have demonstrated differences in composition, mechanics, and inflammation between plaque rupture and plaque erosion, suggesting that considerably different etiologies are at play in each phenotype. The inflammatory phenotype and biomechanical environment of plaques with erosion most closely resemble those of thick-cap fibroatheromas, a stable phenotype, and differ significantly from ruptured plaques and TCFAs. With increasing mechanical strain, there was no corresponding increase in inflammation in plaque erosion, whereas in contrast, inflammation and mechanical strain are closely associated in plaque rupture (Figures 5–8). We also noted numerous similarities between plaque rupture and its vulnerable phenotype the TCFA, compatible with current understanding regarding the characteristics of unstable plaque development. Both eroded and thick-cap fibroatheroma phenotypes express relatively higher levels of smooth muscle cells and have thick, intact media, while the opposite is true for both ruptured plaques and TCFA. In total, eroded plaques have a phenotype that is more closely aligned with that observed in thick-cap fibroatheroma (a stable phenotype). These provocative findings are consistent with the concept that inflammatory processes are necessary for progression to plaque rupture. However, they also suggest that alternative mechanisms must be considered to understand and prevent plaque erosion. These findings raise the possibility that some thick-cap fibroatheroma plaques (i.e., a stable phenotype) may be “vulnerable” for progression to erosion.

The divide in etiologies is most apparent in the relationship between mechanical strain and inflammatory markers. With increasing strain, CD68, MMP1, MMP9, and apoptosis all increased in TCFAs and ruptured plaques. In thick-cap fibroatheromas and plaque erosions, however, we observed no such association between inflammation and strain. Macrophage accumulation and MMP1 expression have been shown previously to be stress and strain sensitive in atherosclerotic lesions [8]. Therefore, their lack of response to mechanics in this subset of lesions, not to mention their lower overall staining, suggests that plaque erosion is not a consequence of the inflammatory processes leading to plaque rupture.

This difference between plaque rupture and plaque erosion may have significant implications for patient care. Whereas plaque rupture is largely an inflammatory disease, plaque erosion appears to be less so. Anti-inflammatory therapeutic techniques used to treat atherosclerotic plaques may not be appropriate to prevent plaque erosion. Two major clinical trials (CIRT and CANTOS) have recently been launched investigating whether anti-inflammatory strategies will reduce cardiac event rates [23], [24]. These trials are motivated by prior studies indicating positive associations between C-reactive protein (CRP) levels and incidence of cardiac events [25]–[27]. While CRP screening may identify patients with inflammation at risk of plaque rupture, these trials may not target patients at risk for plaque erosion. While much is still unknown about the exact mechanism of erosion, our data suggest that its etiology differs very significantly from that of the current prototypical “vulnerable plaque” and as such may require a different approach for prevention and treatment.

One hypothesis warranting further investigation is the role of endothelial apoptosis and denudation in erosion. Previous studies have established this as a likely mechanism for erosion [4], [5], but the cause of endothelial apoptosis, especially if not inflammatory as suggested by the present study, remains elusive. We observed significantly less apoptosis in plaque erosion than in rupture or TCFAs when considering the whole vessel, and when focusing specifically on the endothelium we saw no significant difference in apoptosis between any of the lesion phenotypes. Denudation resulting in endothelial cell loss in plaque erosions could explain this lack of difference in positive TUNEL staining between phenotypes. However, we observed significantly more Factor VIII, a marker of endothelium, in erosion specimens. This may be biased by our study methodology: we excluded regions where thrombus adhered to the wall because of the impossibility of distinguishing whether its source was endothelium or thrombus [28]. But, regions where thrombus has adhered to the wall are precisely the sites where denudation is expected. Despite this limitation, these findings are perplexing. If denudation occurs because of a dysfunctional, apoptotic endothelium, we would still expect more apoptosis and fewer endothelial cells near the lumen in erosion. If apoptosis leading to denudation is not the explanation, further refinement of this etiological hypothesis is warranted.

We recently investigated the role of hemodynamics in plaque erosion, motivated by the idea that highly shearing flow might denude endothelium [29]. Using data from patients with acute cardiac events due to plaque erosion, we found no evidence of flow patterns likely to damage endothelium. In the present study, we lack data on the 3-dimensional morphology of the lesions necessary to reconstruct the flow field across the lesion, and thus we can only assume a constant pressure normal to the surface of the lumen. However, we also did not find elevated endothelial denudation in plaque erosion. Although thrombosis without rupture into the necrotic core is certainly a real phenomenon, together, these findings suggest that the term “erosion” could be a misnomer.

Another noteworthy difference between plaque erosion and rupture is the presence of smooth muscle cells in the intima and media. We observed significantly thicker media in eroded plaques and thick-cap fibroatheromas when compared to ruptures. Loss of media in advanced atherosclerotic plaques is an established phenomenon [30], [31], but does not seem to be at play in either of these plaque phenotypes. Smooth muscle cells are essential for pathological intimal thickening to occur [3], [32] and so their loss in vulnerable and ruptured plaques likely sets up a different biomechanical environment compared to plaques with erosion.

An established family of characteristics determining risk for plaque rupture is the composition and morphology of lesions [33]–[36]. Factors like the size and shape of necrotic core and fibrous cap thickness affect the distribution of mechanical stresses, which leads to plaque vulnerability and potentially then to rupture. In the present study, plaque burden and eccentricity, as well as proportion of fibrous and lipid/necrotic tissue, were not different between phenotypes. Radius, lesion area, percent cellularity, and percent calcification were different between erosion and rupture or TCFA, but other than calcification, which may play a role in lesion stability [37], [38], these are not established characteristics of plaque vulnerability. That morphology and composition were not, on the whole, different between plaque phenotypes strengthens our conclusion that inflammatory response to biomechanics is a key differentiator between plaques that rupture and plaque erosions.

Because we used pressure-fixed tissue as the basis for our mechanical modeling, we were only able to compute the relative distribution of stress and strain, not the absolute magnitude of either value [8], [13]. Other studies modeling plaque rupture have attempted to discern a threshold at which a fibrous cap breaks [7], [39], [40]. A similar analysis is not possible with our technique. For plaque erosion, no gross tissue damage is expected. Unfortunately, because we do not have the ability to calculate absolute magnitudes, we cannot evaluate how levels of stress and strain directly compare between rupture and erosion. To compare vessels, we have normalized stresses and strains to each vessel's median values in order to account for different ranges of values from section to section. Although we cannot say that a high value in one vessel is identical in magnitude to a high value in another, our histogram analysis technique of stress and strain excursions ensures we are always comparing values that are, locally, the highest and lowest among subjects. Additionally, we have compared the median stress and strain values among all groups and found no statistically significant difference (p>0.58) in the overall magnitude of stress and strain between specimens. We assume that the magnitude of residual stress is small compared to the magnitude of incremental stress from our model, and therefore we assume that the relative range of computed stresses are approximately comparable from specimen to specimen. That said, future modeling studies based upon tissue specimens with known residual stress values would be an important confirmation of this work.

Additionally, erosion is not thought to be an acute event [41]. In many cases, a mural thrombus accumulates over a period of approximately a week [41], in contrast to rupture, where thrombosis is typically much more acute. As this thrombus organizes and heals in subjects with erosion, the vessel remodels and the lumen reshapes. Consequently, a histological cross-section of a vessel at the onset of plaque erosion may reveal different information than a cross-section from the time of death. With no present way to detect the initiation of plaque erosion in humans, we are limited to studying autopsy specimens until our understanding of the phenomenon improves. Without such temporal data, we cannot separate whether tissue mechanics led to the up-regulation of the protein markers examined in this study or whether such markers induced vessel remodeling, yielding the mechanical environment of each vessel. Teasing out the complex process of causality between mechanics and biochemistry (which may well involve a feedback loop between the two processes) will be an important task for future research into atherosclerosis.

There is great need for means to detect and treat plaque erosion, particularly in the care of heart disease in women. In this study, we have shown that the inflammatory response to the mechanical environment in atherosclerotic plaques is characteristically different between plaque rupture and plaque erosion. Ruptured plaques and TCFAs have a similar inflammatory phenotype associated with mechanical strain heterogeneity. In contrast, eroded plaques have less inflammation and a mechanical strain profile most closely resembling thick-cap fibroatheromas, traditionally considered a stable phenotype. It is likely that management and treatment for patients experiencing plaque erosion will require an entirely different approach than the current paradigm for those with plaque rupture. As the incidence of plaque erosion differs considerably between men and women, patient-specific factors will likely play a major role in future treatment options for patients with acute coronary syndromes.

## Supporting Information

Table S1 Composition and morphology. For each subject, we quantified the average radius in mm (Radius_mm), the cross-sectional area containing tissue in µm^2^ (Area_um2), the average thickness of the media in µm (Media_thick_um), the average thickness of the lesion in µm (Lesion_thick_um), the fraction of pixels within the inner and outer borders of the vessel segmented as background (Background_percent), calcification (Calcium_percent), fibrous tissue (Fibrous_percent), cellular tissue (Cellular_percent), or lipid/necrotic core (Lipid_percent), the percent of the area inside the internal elastic lamina containing lesion (Plaque_burden), and the eccentricity (distance between lumen centroid and IEL centroid, normalized to vessel radius, Eccentricity_normalized_to_radius).
(CSV)

Table S2 Immunohistochemical staining and segmentation. For each subject, we segmented the number of pixels exhibiting positive staining (columns with suffix “_Pos”) as well as the total number of pixels between the inner and outer borders of the vessel cross-section (columns with suffix “_Tot”). Neither pixel count includes pixels from regions that were identified as not containing tissue (such as regions where tissue detached from the slide or regions of necrotic core where no tissue remained after histological processing). The fractional amount of positive pixels to total pixels is summarized in columns with suffix “Pct”. Sections that were not included in the analysis due to extreme artifact such as the section detaching from the slide entirely were marked as having a Total pixel count of zero and a Percent of NaN (not a number). Stains performed were CD31/CD34 (CD3134), CD68 (CD68), Factor VIII (F8), MMP1 (MMP1), MMP9 (MMP9), Smooth Muscle Actin (SMA), and TUNEL (Apoptosis).
(CSV)
